# Predicting and reasoning about replicability using structured groups

**DOI:** 10.1098/rsos.221553

**Published:** 2023-06-07

**Authors:** Bonnie C. Wintle, Eden T. Smith, Martin Bush, Fallon Mody, David P. Wilkinson, Anca M. Hanea, Alexandru Marcoci, Hannah Fraser, Victoria Hemming, Felix Singleton Thorn, Marissa F. McBride, Elliot Gould, Andrew Head, Daniel G. Hamilton, Steven Kambouris, Libby Rumpff, Rink Hoekstra, Mark A. Burgman, Fiona Fidler

**Affiliations:** ^1^ MetaMelb Research Initiative, School of Ecosystem and Forest Sciences, University of Melbourne, Parkville 3010, Australia; ^2^ MetaMelb Research Initiative, School of Historical and Philosophical Studies, University of Melbourne, Parkville 3010, Australia; ^3^ Centre of Excellence for Biosecurity Risk Analysis, School of BioSciences, University of Melbourne, Parkville 3010, Australia; ^4^ School of Psychological Sciences, University of Melbourne, Parkville 3010, Australia; ^5^ Centre for the Study of Existential Risk, University of Cambridge, Cambridge, UK; ^6^ Martin Conservation Decisions Lab, Department of Forest and Conservation Sciences, University of British Columbia, Vancouver, Canada; ^7^ Centre for Environmental Policy, Imperial College London, London, UK; ^8^ Department of Pedagogical and Educational Sciences, University of Groningen, Groningen, The Netherlands

**Keywords:** replication, forecasting, expert judgement, meta-research, metascience, mixed methods

## Abstract

This paper explores judgements about the replicability of social and behavioural sciences research and what drives those judgements. Using a mixed methods approach, it draws on qualitative and quantitative data elicited from groups using a structured approach called the IDEA protocol (‘investigate’, ‘discuss’, ‘estimate’ and ‘aggregate’). Five groups of five people with relevant domain expertise evaluated 25 research claims that were subject to at least one replication study. Participants assessed the probability that each of the 25 research claims would replicate (i.e. that a replication study would find a statistically significant result in the same direction as the original study) and described the reasoning behind those judgements. We quantitatively analysed possible correlates of predictive accuracy, including self-rated expertise and updating of judgements after feedback and discussion. We qualitatively analysed the reasoning data to explore the cues, heuristics and patterns of reasoning used by participants. Participants achieved 84% classification accuracy in predicting replicability. Those who engaged in a greater breadth of reasoning provided more accurate replicability judgements. Some reasons were more commonly invoked by more accurate participants, such as ‘effect size’ and ‘reputation’ (e.g. of the field of research). There was also some evidence of a relationship between statistical literacy and accuracy.

## 1. Introduction

Over the last decade, several large-scale projects have attempted to replicate the findings of research in preclinical medicine, economics and psychology. The results of these studies have arguably brought the evidence base in those disciplines into question [1–5]. This ‘replication crisis’ arises in many areas of science, undermining the support for decision-making and public trust in science. Unfortunately, the extent of these problems has not yet been fully evaluated. Outside of large-scale replication studies such as those cited above, attempts to directly replicate original research are published only rarely [6,7]. Inappropriate funding and publishing incentives [8] offer researchers little motivation to engage in replication studies [9]. Replication studies also face logistical challenges, including obtaining requisite methods, materials and data from the original researchers [10,11].

Given the challenges of conducting replication studies, judgements from people about the replicability of research may offer a cost-effective alternative for evaluating the reliability of the evidence base. Those that are considered unlikely to be replicable could then be the target of actual replication studies [12]. Fortunately, previous research has shown that, in the aggregate, people are quite good at predicting research replicability in the social and behavioural sciences. In psychology, Dreber *et al.* [13] found a prediction market correctly classified 71% of studies as being replicable (or not) compared with 58% for a simple survey. In social science, predictions about the replicability of 21 experiments published in *Nature* and *Science* achieved 86% classification accuracy, i.e. aggregated estimates were on the ‘right’ side of 50% in 18 of 21 studies, for both a prediction market and simple survey [14]. In experimental economics, the classification accuracy equalled the replication rate of 61% for both the prediction market and the simple survey [15]. And again, in psychology (Many Labs 2), prediction market classification accuracy was 75% and the simple survey was 67% [16]. Simple surveys and prediction markets provide similar estimates, but survey predictions tend to be less extreme and, therefore, perform less well, when predictions are reasonably good to begin with. What is more, even laypeople (those without a PhD or other equivalent training in research methods) have an above-chance prediction accuracy [17,18].

In the Cancer Biology Replication Project, Errington *et al.* [11,19] planned replication studies for 193 experiments. Of these, none were described in enough detail in the original paper to design replication protocols without clarifications from the original authors, making predicting replicability difficult. Forecasts of the replicability of five studies in preclinical cancer research [20], based on replication studies conducted by Errington *et al.* [2], were overly optimistic. Participants tended to believe that original findings *would* successfully replicate, i.e. that the replication studies would obtain a statistically significant effect in the same direction as the original study. For experts, 73% of their forecasts indicated replication success, when *none* of the five replication studies successfully replicated under the ‘significance’ criterion.

Individual performance in the above studies varies and unfortunately, it is difficult to know in advance who will be an accurate judge or forecaster [21]. In one of the few, large, longitudinal studies of expert judgement, Tetlock [22] found that professional background and status do not translate to accuracy, but the way experts think and reason matters. We continue to seek different markers of expertise because identifying the best forecasters could guide decisions about which experts to consult in the first place, research in this field is highly context-dependent, and it's not always domain-level expertise that turns out to be important. For example, expertise in statistical analysis and methodology may be valuable when experts are evaluating the replicability of research papers on the fringes of their narrow substantive expertise, as they are doing in this study, and as they effectively often do when tasked with peer review. Many of our research questions here are motivated by this, and other, questions in the Judgement and Decision Making literature. For example, we test participants' prior knowledge on topics related to the main task of assessing papers, hypothesizing that performance on the quiz (a proxy for expertise) might lead to better predictions. This connects with Cooke's theory and the classical method of weighting different experts according to their performance on calibration or seed questions [23].

In exploratory analyses, we investigate possible relationships between the demographics, experience and psychometric test scores of judges and the characteristics of their judgements, namely, prediction accuracy and the propensity to update their judgements in light of new information, which is a characteristic of good, flexible forecasting [22]. These analyses remain exploratory because, despite decades of related research on expert judgement, the field still lacks clear theories or hypotheses about most of these questions.

One characteristic that *may* point to more or less accurate judgements is confidence. While evidence suggests that more confident *individuals* are no more accurate on average [21], there is some evidence that judgement-level confidence, measured in various ways, contains potentially useful information [24]. However, even if some measures of confidence provide information about which judgements might be more accurate relative to other judgements, it does not help us decide in advance which judge to consult. Aggregated judgements from multiple people are almost always preferable to relying on a single individual (the so-called Wisdom of Crowds).

**Figure 1.**
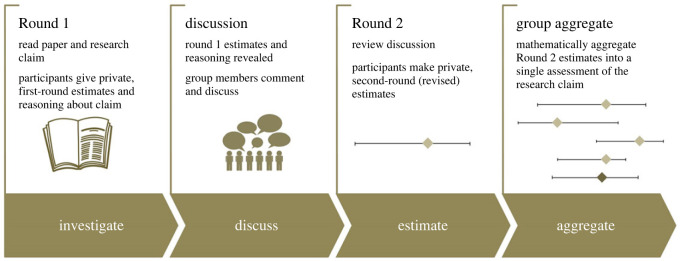
Overview of the IDEA protocol, as adopted in the repliCATS project. First published by Fraser *et al.* [31].

This logic underpins our adoption of a structured protocol for eliciting judgements from groups, called the ‘IDEA protocol’ (‘investigate’, ‘discuss’, ‘estimate’ and ‘aggregate’) [25,26]. The protocol includes techniques for avoiding anchoring and dominance effects and reducing the overconfidence of assessors, including eliciting quantitative judgements in an interval format. That is, participants are prompted to provide their (i) lower bound, (ii) upper bound and (iii) best estimate of the quantity or probability being elicited from the experts (such as the probability that a social science claim would successfully replicate) [27–29]. It combines the judgements of individuals using mathematical [30] rather than behavioural aggregation approaches, so group members are not forced to agree on a consensus judgement. A crucial aspect of the IDEA protocol is that it incorporates a ‘Discussion’ phase, allowing diverse group members to share information and interrogate each others' judgements. This phase is particularly advantageous when experts have private information that would be valuable to a group judgement, as is likely the case in this context, given the role of private insights in previous replicability predictions [13]. A summary of the IDEA protocol as adapted in this research is in figure 1.

The IDEA protocol integrates judgements from multiple people and provides insight into how assessors go about making judgements, a topic that has not been systematically investigated in other studies on forecasting replicability. Indeed, we know little about how reviewers go about assessing scientific manuscripts in general [32,33]. In this paper, we describe how we collect and analyse the reasoning and justifications that participants provide alongside their quantitative predictions of replicability.

The main purpose of this paper is to better understand the judgements people make about the reliability of research, and how they reason around their assessments. In doing so, the paper examines some correlates of predictive accuracy, particularly which, if any, of our demographic, confidence and expertise measures correlate with the ability to predict the outcomes of replication studies. Aside from looking at relationships with accuracy, it provides a rich data source to explore the reasons participants provided for their assessments of claims. It also allows us to build a proxy measure for performance, a breadth-of-reasoning score based on our qualitative analysis. Our intuition is that this may be a useful proxy measure for assessments of the reliability of claims. Since these techniques were developed in the course of this project, they should be considered exploratory and provisional. Nonetheless, we will describe some of the signals derived from this ‘breadth of reasoning’ score as potentially useful examples of characteristics of good reasoning and judgements.

## 2. Hypotheses and exploratory questions

To address the research aims described above, specific research questions were developed (table 1). In particular, we developed four pre-registered ‘confirmatory’ hypotheses that predict an effect in a particular direction. ‘Exploratory’ research questions were also flagged in the pre-registration but were not accompanied by such clear directional statements. Those listed as ‘not pre-registered’ did not appear in the pre-registration at all.

**Table 1.** Hypotheses and research questions.

topic	level	pre-registration status	research question/hypothesis
accuracy	participant	confirmatory	Hypothesis 1: Round 2 judgements (post-discussion) will be more accurate than Round 1 judgements (pre-discussion).
accuracy	participant	confirmatory	Hypothesis 2: Those who perform well on a quiz testing familiarity with previous research and concepts related to assessments of replicability will be more accurate on the main task (both rounds, but in particular, Round 1).
accuracy	participant	exploratory	Question 1a: What are the demographic, experience and psychometric characteristics of more accurate judges?
updating	participant	exploratory	Question 1b: What are the demographic, experience and psychometric characteristics of those who update their judgements more between rounds?
updating/accuracy	participant	exploratory	Question 2: Are those who update their judgements more between rounds more accurate than other participants in Round 2 (after discussion, across all claims)?
updating	participant	confirmatory	Hypothesis 3: Those who update their estimates (best estimates and bounds) more between rounds will have (i) lower average claim-level expertise ratings and (ii) lower average claim-level understanding ratings.
accuracy/expertise, understanding, precision	judgement	confirmatory	Hypothesis 4: Judgements made on particular claims (i.e. not aggregated across claims for that participant) will be more accurate when associated with (i) higher claim-level expertise ratings, (ii) higher claim-level understanding ratings and (iii) more certain (narrower) intervals.
improvement/expertise, understanding	participant	not pre-registered	Question 3: Do those with lower expertise and understanding *across claims* (signalled by (i) lower average claim expertise ratings and (ii) lower average claim understanding ratings) improve more after discussion? (become more accurate)
reasoning breadth/accuracy	participant	not pre-registered	Question 4: Are those who use a greater breadth of reasons to support their assessments more accurate?
reasoning categories/accuracy	participant	not pre-registered	Question 5: Which reasoning categories are more commonly used by more accurate participants?

## 3. Material and methods

The pre-registration is publicly available through the Open Science Framework (OSF) (https://osf.io/5rj76). Materials and anonymized data, including R code used for analyses, can be found on the OSF page for this study (https://osf.io/pj3v8) [34]. Although data had been collected before pre-registration, the author of the pre-registration (BW) was blinded to the data and any preliminary analyses before uploading the pre-registration, and was not directly involved in data collection. This study was approved as part of the larger repliCATS project (Collaborative Assessment of Trustworthy Science) [31] by the ethics board at the University of Melbourne (Ethics ID: 1853445).

### 3.1. Participants

The study was undertaken as part of a larger 2-day workshop to assess the replicability of claims for the SCORE (Systematizing Confidence in Open Research and Evidence) programme (https://www.darpa.mil/program/systematizing-confidence-in-open-research-and-evidence). The main aim of the workshop was to obtain confidence scores related to the replicability of social and behavioural science claims, from groups, using the IDEA protocol. The workshop was scheduled alongside the Society for the Improvement of Psychological Science Conference 2019 in Rotterdam, Netherlands. We offered participants bursaries (AUD400 if from the UK/EU and AUD1000 if outside of the UK/EU) to cover their travel expenses, allowing them to attend our workshop as well as the conference. Our resulting study sample consisted of those who applied for the bursaries. All participants were over 18 years of age. In total, 156 participants were recruited to attend the larger workshop, of whom 25 were randomly assigned to participate in this study, and allocated to five groups. The remaining 20 groups (not reported on here) assessed claims for which there were no existing replication study outcomes at the time. A sample of these was later selected for replication.

Before attending the workshop, participants completed demographics questions that were used to allocate participants into groups, aiming to balance gender, region (European/non-European resident) and education (students versus non-students) (see electronic supplementary material for further information).

### 3.2. Sampling plan

In total, this study involved *N* = 25 participants in five IDEA groups (5 groups × 5 participants), each assessing 25 questions with ‘known-outcome’ claims (i.e. social and behavioural science claims that were subject to at least one replication study) (electronic supplementary material). The sample size was determined by considering both logistics and the reliability of Brier scores. Brier scores measure the long-run accuracy of participant forecasts. Previous analyses conducted by members of our team indicated that Brier scores are unstable if fewer than 20 binary outcome questions are answered by each participant. Other research teams involved in predicting the outcomes of geopolitical events [35] have also previously specified a minimum of 25 questions for reliability. We settled on a design that required each participant to answer 25 binary outcome questions (i.e. to assess the replicability of 25 claims). This also accounted for possible variation in the difficulty of claims. We stopped collecting data for this project at the end of our 2-day workshop (stopping rule determined by pre-allocated workshop time).

### 3.3. Materials

We selected 25 claims to be evaluated from a database of 341 claims compiled from the major replication projects (pre-2019) from the social and behavioural sciences. This was provided to us (to FST) by the Centre for Open Science. Using this database of known-outcome claims, we first applied some exclusion criteria. Specifically, the claims were reduced from the original set using the following rules:

(i) Selected claims were from Many Labs 1 [3], Many Labs 2 [5] and Many Labs 3 [36], the Social Sciences Replication Project (*Nature* and *Science*) [14] or the original Reproducibility Project Psychology [4].
(ii) The replication had at least 90% power to detect an effect 75% of the size of that seen in the original study (calculated using Fisher Z transformed correlation coefficients).
(iii) Ten claims from the sample set were excluded as having been subject to media attention (potentially disclosing the outcome of the replication study to the participants). Details of excluded studies are in electronic supplementary material.

Before attending the workshop, participants completed their consent and demographics forms, together with a short quiz in Qualtrics, testing their knowledge and understanding of statistical concepts, meta-research and other items relevant to the main task. They were also asked to access our training materials (including a glossary, videos and links to short e-courses; https://osf.io/a89nc/) to familiarize themselves with relevant meta-research and statistical concepts relevant to assessing claims.

**Figure 2.**
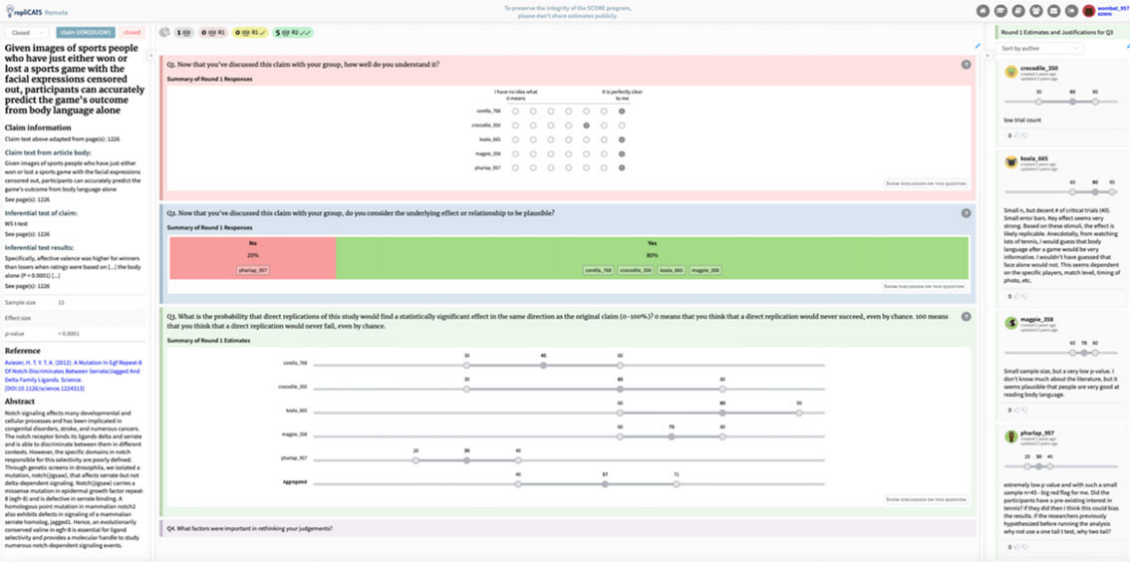
The online repliCATS platform that participants used to evaluate research claims.

To evaluate claims, participants answered a set of questions on an online repliCATS platform (figure 2) we developed for the SCORE programme [31,37]. See electronic supplementary material for a full list of questions.

### 3.4. Procedure

Before data collection, anonymized usernames, groupings and claims were imported into our online repliCATS platform. In a face-to-face setting, but using the online platform, each of the five groups evaluated the same 25 ‘known-outcome’ claims across 2 days.

Groups were facilitated by members of our research team (F.M., A.M.H., R.H., M.M. and L.R.) who were blinded to the claims for assessment (i.e. they did not know the outcomes themselves). We were concerned that participants might recognize some of the claims and either share the ‘answer’ or unduly influence others in the discussion, so facilitators set some ground rules at the start of the workshop, asking participants to abstain from the discussion if they were confident that they ‘knew’ the outcome. Facilitators were briefed beforehand and provided a procedural checklist of items to explain to participants, to promote consistency between groups. Specifically, they were given guidelines for prompting discussion, considering counterfactuals, encouraging a range of opinions to be expressed, together with guidelines around time to spend on each claim (approx. 30 min), tips for answering and interpreting each question on the platform, and training materials, e.g. to help participants interpret common statistics they may see reported in the papers they were evaluating (see OSF materials for further information).

After logging on to the platform, participants saw a claim summary that included key statistics, including details of the inferential test used in the paper, the effect measure, effect size and sample size (figure 2), the abstract of the paper from which the claim was extracted and a link to the full paper. Following the IDEA protocol, participants were first invited to *investigate* the claim privately.

In the main quantitative task for Round 1, participants were asked to assess the probability that each claim would successfully replicate (i.e. a replication study would find a statistically significant result in the same direction as the original study), by providing a three-point interval judgement (lower bound, upper bound, best estimate). They were also asked to assess how well they understood the claim (1–7), whether they considered the underlying effect or relationship to be plausible (binary), and to rate their expertise in the field that the claim pertained to (off-platform, 1–7).

In the main qualitative task for Round 1, participants were asked to enter the reasoning behind their judgements into the platform. The schedule of the 2-day workshop allowed participants on average around 15 min to read through the claim and provide their private Round 1 judgements and their reasoning.

Following the submission of the Round 1 answers, group members' anonymous estimates and reasoning were revealed, and the group could compare and *discuss* (face to face) their estimates, share information and cross-examine reasoning and evidence. The facilitated discussion was also scheduled to last, on average, around 15 min. After discussion, group members individually entered a second, final, private *estimate.* Round 2 estimates were combined using mathematical *aggregation*.

There was some flexibility around the order and manner in which claims were assessed between groups, e.g. some groups preferred to complete Round 1 in batches of three to five claims before moving on to the discussion/Round 2 phase, whereas others worked through the full IDEA process, claim by claim. At the start of the workshop, claims were listed on the platform in the same order for all groups. We did not set rules about working through the claims in any particular order. Throughout the process, the ordering of claims on the repliCATS platform changed, sorted by which round they were in, with claims still in Round 1 followed by claims in Round 2. This helped individuals track the progress of claims they had completed [31,37].

### 3.5. Measures

#### 3.5.1. Accuracy

We calculated the accuracy of participants’ best estimates (Rounds 1 and 2 probabilities) using a distance (error) measure from the ‘true value’. In this case, the ‘true value’ is a binary outcome for whether or not the claim is replicated (0, 1).

For accuracy measures at the judgement level, we used a simple distance measure, i.e. the absolute error between the estimated probability of replication (converted from percentage to proportion) and 0 or 1.

For participant-level and claim-level accuracy, the distance measure was combined into Brier scores [38] to measure the long-run accuracy of individual participants over different claims, and the accuracy of different participants on individual claims, using the following:

3.1 $$\mathrm{BS}=\frac{1}{N}\sum_{c=1}^{N}{\left(f_{c}-o_{c}\right)}^{2}.$$

In which *f_c_* is a participant's prediction of replicability, *o_c_* is the actual outcome of the replication attempt at claim *c* (*o_c_* is either 0 or 1: did the claim successfully replicate or not) and *N* is the number of predictions the participant made. Brier scores are a commonly used proper scoring rule for evaluating probabilistic forecasts. They can take values from 0 (perfectly accurate) to 1 (perfectly inaccurate).

#### 3.5.2. Precision (interval width)

For precision, we calculated an interval width between 0 and 1 for each interval judgement by subtracting the lower bound from the upper bound.

#### 3.5.3. Updating (shifting)

We measured the shift in estimates between rounds, separately for best estimates and interval widths |Round 1–Round 2|.

#### 3.5.4. Improvement (in accuracy)

We measured the improvement between rounds as a reduction in Brier scores, Round 2 BS–Round 1 BS. Since this variable is measured as a reduction in error, negative values signify improvement.

#### 3.5.5. Prior knowledge

We approximated prior knowledge using performance on a quiz compiled by the repliCATS elicitation team (B.W., A.M.H. and V.H.), testing familiarity with previous research and concepts related to assessments of replicability (statistical and meta-research concepts). For example: ‘The *p*-value gives the probability of obtaining a significant result whenever a given experiment is replicated’ (response options: true/false/I do not know). The quiz contained 22 questions, and participants received one point for each correct answer. Quiz scores ranged from 0 to 22.

#### 3.5.6. Expertise (understanding, confidence)

We approximated a participant's expertise *on each claim* (judgement level) with three measures: *claim-level expertise* (self-reported, ordinal variable scored 1–7), *claim-level understanding* (self-reported, ordinal variable scored 1–7) and *claim-level interval width* (i.e. the interval width measure, described above). The latter two measures were collected for both Rounds 1 and 2.

In addition, for each participant (participant level), we approximated general expertise *across all claims*, by averaging within-participant claim-level responses above, giving us a measure of *overall claim expertise* (i.e. self-reported, ordinal variable scored 1–7, averaged for the participant) and *overall claim understanding* (i.e. self-reported, ordinal variable scored 1–7, averaged for the participant). We acknowledge the long-debated concerns with averaging ordinal scales (assuming equidistant intervals from 1 to 7) to gain a single score per participant, and so interpret those measures with caution.

### 3.6. Quantitative analysis

Due to the groupings in the study design (i.e. judgements made by each individual, judgements by different individuals on a given claim, and multiple individuals in an IDEA group), we expected a cluster effect in Round 2 judgements. We opted for modelling that accounts for this. We fitted linear mixed models in R [39] using the nlme [40], lme4 [41] and lmerTest [42] packages, and accounted for the variation arising from different groups as well as from repeated measures (25 judgements per participant), by including these variables as random effects. For the judgement-level models, the ‘claim’ variable was also included as a random effect, but this was not necessary for the participant-level models, where judgements are aggregated across claims. Degrees of freedom in the mixed-effects models were calculated using Satterthwaite's formula. In addition to the pre-registered modelling, we separately performed planned comparisons and correlation analyses, and the results were consistent with the modelling (see electronic supplementary material).

For Hypothesis 1, we calculate and plot 95% confidence intervals (CIs) to enable ‘inference by eye’ comparisons [43] to infer differences between groups (rounds). That is, differences are statistically significant at the equivalent of *α* <0.05 when there is less than 25% overlap between groups' 95% CIs. We also report the *p-*value.

For Hypotheses 2–4, we calculated *p-*values and plotted standardized model coefficients with 95% CIs. Our inferences and conclusions consider both pieces of information.

No data were excluded from confirmatory analyses. Missing data points were treated as NAs, participants with NA data were included in the analyses and sample sizes were adjusted accordingly for those variables.

*Hypothesis 1: Round 2 judgements (post-discussion) will be more accurate than Round 1 judgements (pre-discussion) (at the participant level).*

We investigated increased accuracy in final judgements by plotting participants’ Average Brier scores and 95% CIs before (Round 1) and after discussion (Round 2).

Additional exploratory analysis: To further test this hypothesis, we also perform a *t*-test for the difference between groups of paired data, treating pre-/post-discussion as pre-/post-test.

*Hypothesis 2: Those who perform well on a quiz testing familiarity with previous research and concepts related to assessments of replicability will be more accurate on the main task (at the participant level).*

We separately modelled individuals' accuracy in Rounds 1 and 2 (Brier scores) as a function of their quiz scores (equations (3.2) and (3.3), respectively). We included a random ‘group’ effect in both Rounds 1 and 2 analyses. Even though the group effect would presumably be less for Rounds 1 than 2, there is still potential for facilitator influence (e.g. explaining instructions, allowing different procedures for assessing claims), different settings (e.g. some groups shared large rooms, which may have been noisier) and other group-level effects (e.g. a particularly pessimistic or optimistic group might affect Round 1 judgements made later in the workshop)

3.2 $$\begin{array}{rl} \mathrm{Round}\_1\_\mathrm{Brier}\_{\mathrm{Score}}_{i}\; & ∼\;N(\alpha_{\;j[i]}+\;\beta_{1}(\mathrm{quiz}\_\mathrm{score}),\sigma^{2})\\ \alpha_{j}\; & ∼\;N(\mu_{\alpha_{j}},\sigma_{\alpha_{j}}^{2})\;\mathrm{for}\;\mathrm{group}\;j=1,\ldots ,J \end{array}$$

and

3.3 $$\begin{array}{rl} \mathrm{Round}\_2\_\mathrm{Brier}\_{\mathrm{Score}}_{i}\; & ∼\;N(\alpha_{\;j[i]}+\;\beta_{1}(\mathrm{quiz}\_\mathrm{score}),\sigma^{2})\\ \alpha_{j}\; & ∼\;N(\mu_{\alpha_{j}},\sigma_{\alpha_{j}}^{2})\;\mathrm{for}\;\mathrm{group}\;j=1,\ldots ,J. \end{array}$$

Additional exploratory analysis: we separately explored if scores on each of the two core components of the quiz (knowledge of statistical concepts and knowledge of meta-research) had the same relationship with accuracy. Here we used Spearman rank correlations and 95% CIs, since this was exploratory, participant level (no need for a claim-level random effect) and we were mainly interested in Round 1 (before discussion when participant groupings are unlikely to affect judgements).

*Exploratory Question 1: What are the demographic, experience and psychometric characteristics of (i) more accurate judges and (ii) those who update their judgements most between rounds?*

In addition to the pre-registered modelling, we also analysed additional participant demographic and background information on; gender, age, education, career stage, technical expertise (i.e. mathematics (generally), quantitative modelling/simulation, statistics, probability, experimental design, risk analysis, forecasting and 1–7), other relevant experience (with direct and/or partial/conceptual replication; forecasting replication studies; meta-research and/or pre-registration, all binary), publications and training (background questions, https://osf.io/pj3v8/.

In the same survey, we also included ten items from the actively open-minded thinking (AOT) test [44], three items from the cognitive reflection test (CRT) [45] and four from the Berlin Numeracy (BN) test [46] (https://osf.io/pj3v8/). Two participants were excluded from these analyses due to not answering those questions.

We explored these relationships using Spearman rank correlations. We also ran planned comparisons using ANOVAs. We report 95% CIs throughout, and precise statistical significance is also indicated with *p*-values. Additional results can be found in electronic supplementary material.

*Exploratory Question 2: Are those who update (shift) their judgements more between rounds more accurate in Round 2 (after discussion, across all claims)?*

We explore this question using separate linear mixed models, modelling participants' accuracy (individual Brier scores across all claims) for each round as a function of how much the participant shifted their judgement after discussion (equations (3.4) and (3.5)).

${\mathrm{Shifting}}_{\mathrm{Best}}$ is defined as | Round 1 best estimate−Round 2 best estimate |. As for all previous models, we include a random group effect.

3.4 $$\begin{array}{rl} & \mathrm{Round}\_1\_\mathrm{Brier}\_{\mathrm{Score}}_{i}\;∼N(\alpha_{\;j[i]}+\;\beta_{1}({\mathrm{Shifting}}_{\mathrm{Best}}),\sigma^{2})\\ & \alpha_{j}\;∼\;N(\mu_{\alpha_{j}},\sigma_{\alpha_{j}}^{2})\;\mathrm{for}\;\;\mathrm{group}\;j=1,\ldots ,J \end{array}$$

and

3.5 $$\begin{array}{rl} & \mathrm{Round}\_2\_\mathrm{Brier}\_{\mathrm{Score}}_{i}\;∼N(\alpha_{\;j[i]}+\;\beta_{1}({\mathrm{Shifting}}_{\mathrm{Best}}),\sigma^{2})\\ & \alpha_{j}∼\;N(\mu_{\alpha_{j}},\sigma_{\alpha_{j}}^{2})\;\mathrm{for}\;\;\mathrm{group}\;j=1,\ldots ,J. \end{array}$$

*Hypothesis 3: Those who update (shift) their judgements most between rounds will have (i) lower mean claim-level expertise ratings and (ii) lower mean claim-level understanding ratings (at the participant level).*

We modelled the difference in participants’ best estimates (equation (3.6)), and the difference in their interval widths (equation (3.7)), between Rounds 1 and 2 as a function of mean expertise and mean understanding averaged across all claims for a given participant.

${\mathrm{Shifting}}_{\mathrm{Best}}$ is defined above. ${\mathrm{Shifting}}_{\mathrm{Interval}\_\mathrm{Width}}$ is defined as | Round 1 interval width −Round2 interval width |. We included a random group effect term to account for group-specific variation.

3.6 $$\begin{array}{rl} {\mathrm{Shifting}}_{{\mathrm{Best}}_{i}}\;∼ & \;N(\alpha_{\;j[i]}+\;\beta_{1}(\mathrm{mean}\_\mathrm{expertise})+\;\beta_{2}(\mathrm{mean}\_\mathrm{understanding}),\sigma^{2})\\ \alpha_{j}\;∼ & \;N(\mu_{\alpha_{j}},\sigma_{\alpha_{j}}^{2})\;\mathrm{for}\;\mathrm{group}\;j=1,\ldots ,J \end{array}$$

and

3.7 $$\begin{array}{rl} {\mathrm{Shifting}}_{{\mathrm{Interval}\_\mathrm{Width}}_{i}}\;∼ & \;N(\alpha_{\;j[i]}+\;\beta_{1}(\mathrm{mean}\_\mathrm{expertise})+\;\beta_{2}(\mathrm{mean}\_\mathrm{understanding}),\sigma^{2})\\ \alpha_{j}\;∼ & \;N(\mu_{\alpha_{j}},\sigma_{\alpha_{j}}^{2})\;\mathrm{for}\;\mathrm{group}\;j=1,\ldots ,J. \end{array}$$

N.B.: Since ‘understanding’ was elicited in both rounds, we needed to choose between Rounds 1 or 2 scores for this ‘shifting’ analysis (not specified in the pre-registration). Although it does not substantively affect results, we opted for Round 1 ‘understanding’ scores here, since coming from a place of low understanding at the start might suggest that participants are more likely to shift.

*Hypothesis 4: Judgements made on particular claims (i.e. not aggregated across claims for that participant) will be more accurate when associated with (i) higher claim-level expertise ratings, (ii) higher claim-level understanding ratings and (iii) more certain (narrower) intervals.*

We modelled the difference between individuals' best estimates and the known replication outcome separately for each round (equations (3.8) and (3.9)). The difference for each round was modelled as a function of individuals’ claim-level expertise, their understanding of the claim for that round, as well as their interval width for that round. In both models, we included random effects for participant, group and claim

3.8 $$\begin{array}{rl} & \mathrm{Round}\_1\_\mathrm{absolute}\_{\mathrm{error}}_{i}\;∼\;N(\mu ,\sigma^{2})\\ & \mu =\;\alpha_{\;j[i],k[i],l[i]}+\;\beta_{1}(\mathrm{claim}\_\mathrm{level}\_\mathrm{expertise})\\ & +\;\beta_{2}(\mathrm{Round}\_1\_\mathrm{claim}\_\mathrm{level}\_\mathrm{understanding})\\ & +\;\beta_{3}(\mathrm{Round}\_1\_\mathrm{claim}\_\mathrm{level}\_\mathrm{interval}\_\mathrm{width})\\ & \alpha_{j}\;∼\;N(\mu_{\alpha_{j}},\sigma_{\alpha_{j}}^{2})\;\mathrm{for}\;\mathrm{group}\;j=1,\ldots ,J\\ & \alpha_{k}\;∼\;N(\mu_{\alpha_{k}},\sigma_{\alpha_{k}}^{2})\;\mathrm{for}\;\mathrm{participant}\;k=1,\ldots ,K\\ & \alpha_{l}\;∼\;N(\mu_{\alpha_{l}},\sigma_{\alpha_{l}}^{2})\;\mathrm{for}\;\mathrm{claim}\;l=1,\ldots ,L \end{array}$$

and

3.9 $$\begin{array}{rl} & \mathrm{Round}\_2\_\mathrm{absolute}\_\mathrm{erro}r_{i}∼\;N(\mu ,\sigma^{2})\\ & \mu =\;\alpha_{\;j[i],k[i],l[i]}+\;\beta_{1}(\mathrm{claim}\_\mathrm{level}\_\mathrm{expertise})\\ & +\;\beta_{2}(\mathrm{Round}\_2\_\mathrm{claim}\_\mathrm{level}\_\mathrm{understanding})\\ & +\;\beta_{3}(\mathrm{Round}\_2\_\mathrm{claim}\_\mathrm{level}\_\mathrm{interval}\_\mathrm{width})\\ & \alpha_{j}\;∼\;N(\mu_{\alpha_{j}},\sigma_{\alpha_{j}}^{2})\;\mathrm{for}\;\mathrm{group}\;j=1,\ldots ,J\\ & \alpha_{k}\;∼\;N(\mu_{\alpha_{k}},\sigma_{\alpha_{k}}^{2})\;\mathrm{for}\;\;\mathrm{participant}\;k=1,\ldots ,K\\ & \alpha_{l}\;∼\;N(\mu_{\alpha_{l}},\sigma_{\alpha_{l}}^{2})\;\mathrm{for}\;\mathrm{claim}\;l=1,\ldots ,L. \end{array}$$

*Exploratory Question 3: Do participants with (i) lower average claim expertise ratings and (ii) lower average claim understanding ratings (across claims) become more accurate after discussion?*

We explore this question using a linear mixed model of the difference in participants' Brier scores between rounds as a function of the mean of their expertise across all claims they assessed as well as the mean understanding across all claims. We include a random-effect accounting for group-level variation (equation (3.10)):

3.10 $$\begin{array}{rl} {\mathrm{Improvement}}_{{\mathrm{Brier}\_\mathrm{Score}}_{i}}\;∼ & \;N(\alpha_{\;j[i]}+\;\beta_{1}(\mathrm{mean}\_\mathrm{expertise})+\;\beta_{2}(\mathrm{mean}\_\mathrm{understanding}),\sigma^{2})\\ \alpha_{j}\;∼ & \;N(\mu_{\alpha_{j}},\sigma_{\alpha_{j}}^{2})\;\mathrm{for}\;\;\mathrm{group}\;j=1,\ldots ,J. \end{array}$$

### 3.7. Qualitative analysis

The reasoning underpinning participants’ assessments of the research claim, particularly about replicability, was collected through free-text responses within the online platform. Three separate text boxes, with specific prompts, were provided in each of the two rounds of the elicitation. Details of these prompts are given in the electronic supplementary material.

Data were analysed using a subset of analytic categories (codes) developed through qualitative content-analysis techniques during the repliCATS project [31,47]. These predefined codes were collected in the ‘Known-Outcome Codebook’, along with inclusion and exclusion criteria, to guide analysts in interpreting text instances with respect to relevant codes. The final codebook included 24 primary analytic categories, including codes that highlighted characteristics of reasoning about both the study design (e.g. sampling practices) and contextual considerations (e.g. journal reputation).

Three analysts (F.M., A.H. and B.M.) independently applied codes to all text units using computer-aided qualitative analysis software, NVivo [48], following training in the codebook and two rounds of calibration. The first round of calibration involved the analysts applying the codes to sample data and then meeting to discuss and resolve differences of interpretation, which were documented in an annotated version of the codebook. The second round of calibration, undertaken after a complete coding of the dataset, involved another meeting to discuss further differences in interpretation. Coders then waited at least two weeks before independently re-coding the data, so that as far as possible they were undertaking a new coding rather than coding from memory. This second coding was regarded as final. The inter-coder reliability (ICR) of the analysts on the final coding was calculated as Krippendorff's alpha score [49]. Details of the final ICR for each of the codes can be found in the electronic supplementary material. For mixed-methods analyses described below, we only included codes for which the ICR score met a predefined minimum value of 0.66 across all three analysts, or a minimum value of 0.50 across all three analysts and at least 0.75 between any two analysts. This was the threshold that had previously been determined for mixed-methods analyses in an earlier paper [47].

*Exploratory Question 4: Are those who use a greater breadth of reasons to support their assessments also more accurate?*

A ‘breadth of reasoning’ score, a potential proxy for performance, was constructed by counting up the number of distinct validated codes that were applied, across all of the textual responses to all questions for a given claim provided by a specific participant.

To investigate whether there might be an association between ‘breadth of reasoning’ and accuracy we ran a linear regression of these scores for each participant and question, against the relevant Round 2 Brier scores. We did not include a random group effect here, as this added complexity interfered with the model fit.

*Exploratory Question 5: Which reasoning categories are more commonly used by more accurate participants?*

A further exploratory question was whether specific codes were used more frequently by more accurate participants. To explore this, we fitted linear regressions to examine the effect of code use frequency on accuracy (i.e. their average Round 2 Brier scores). We then plotted the regression coefficients and associated 95% CIs for each code to produce a measure and visualization of that code's association with accurate patterns of use.

Finally, we scrutinized the coding to seek further insight into processes described by the quantitative hypotheses. Examples of such statements are provided below and should be considered as illustrative.

The ICR threshold for mixed-methods analyses and the codebook used for this analysis were determined before analysis began. However, the qualitative methods described here were not subject to pre-registration. Furthermore, analysis for the exploratory question about the association of the application of specific codes to accurate participants was not determined until after coding had been completed. As such, all of the results of the qualitative analysis presented below should be considered exploratory and provisional.

## 4. Results and discussion

Participants identified as nationals from 13 different countries, collapsed into regions to protect anonymity. They had attained high levels of education and relevant experience for the task at hand, namely, in statistics and/or quantitative research methods (table 2). They were mostly graduate students and early career researchers in psychology. They performed relatively well on the quiz that tested their knowledge of statistical concepts and meta-research.

**Table 2.** Summary of demographic and expertise characteristics of participants. Age elicited as a range, with range midpoints used for descriptive statistics. Means and standard deviation of range midpoints, scores and scales are reported here, together with counts for categorical data.

characteristic	descriptive statistics	
age	mean 34.4 (s.d. 9.7)	
gender	18 female, 7 male	
region (based on nationality)	1 Asia, 1 Africa, 11 Europe, 10 North America, 1 Oceania, 1 South America	
education (highest attained)	9 doctorate, 9 masters, 7 undergraduate	
degree discipline (highest attained)	1 arts/humanities, 1 engineering, 6 science, 17 social science	
career stage	1 undergrad, 15 graduate students, 6 early career, 1 mid-career, 1 senior	
current occupation	23 academia, 1 private sector, 1 public sector	
field of work/interests	1 economics/psychology, 1 education/psychology, 1 physiology, 21 psychology, 1 psychology/sociology	
number of publications (total)	*No. publications*	*count*
	0	9
	1	3
	2	1
	3	2
	4–10	3
	11–20	3
	21–50	3
	51+	1
number of publications (research methods/statistics)	*No. publications*	*count*
	0	20
	1	2
	2	2
	11–20	1
courses taken in statistics and/or quantitative research methods	*No. courses*	*count*
	2	1
	3	2
	4	4
	5	3
	6+	15
teaching experience in statistics and/or quantitative research methods	7 none, 11 some, 7 lots	
technical expertise (scale converted to 0–5)	maths (median 2, mean 2.24, s.d. 0.66)	
	modelling (median 2, mean 1.76, s.d. 1.2)	
	statistics (median 3, mean 3.04, s.d. 0.35)	
	probability (median 2, mean 2.24, s.d. 0.78)	
	experimental design (median 3, mean 3.2, s.d. 0.65)	
	risk analysis (median 0, mean 0.48, s.d. 0.92)	
	forecasting (median 0, mean 0.44, s.d. 0.77)	
experience: editorial board (checkbox)	6 indicated yes	
experience with replication studies (combined direct and partial/conceptual checkboxes)	18 indicated yes	
experience: meta-research (checkbox)	9 indicated yes	
experience: pre-registration (checkbox)	18 indicated yes	
quiz score (max 22 points)	mean 13.4 (s.d. 2.8)	

**Figure 3.**
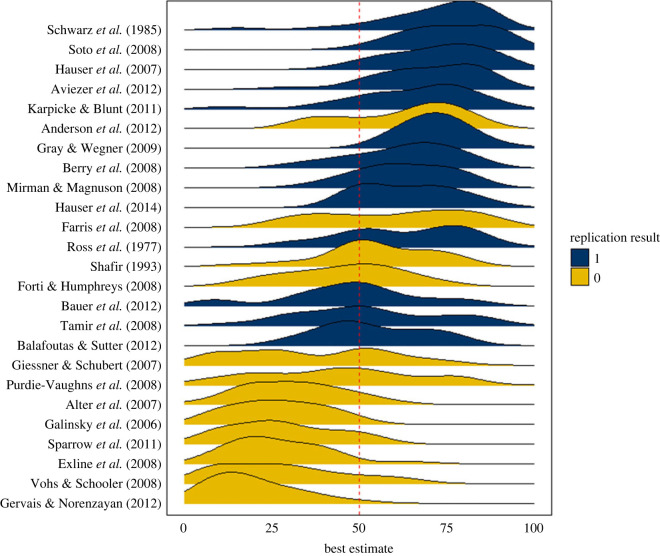
Smoothed distribution of participants' best estimates of replicability for each of the 25 research claims (studies), ordered by descending mean best estimates. Claims that were successfully replicated are shown in blue, and claims that did not successfully replicate are shown in yellow. Best estimates greater than 50% indicate that the participant predicted the claim would successfully replicate, and less than 50% indicate that the participant predicted the claim would fail to replicate.

Participants’ aggregated (average) judgement achieved 84% classification accuracy when predicting the replicability of social and behavioural science research claims included in this study. That is, for the claims participants evaluated, the average probability judgement was greater than 0.5 on those that successfully replicated, and less than 0.5 on those that did not 84% of the time (figure 3 summarizes participant judgements per claim, full references to papers containing each claim can be found in the electronic supplementary material).

For the qualitative data, there were 1893 individual non-blank text responses out of a possible maximum of 3750 (three responses in each of two rounds for the 25 participants for each of the 25 claims assessed). It was not compulsory to complete the text boxes. For the primary target question on direct replication, there were non-blank responses 84.3% of the time in Round 1 (527 responses from a maximum possible 625), while in Round 2 the figure was 60.0% (325 responses from a maximum possible 625). These text responses were typically short. For the target question on direct replication, non-blank responses ranged from one word ('unchanged') to 135 words. The shortest non-blank Round 1 response to this question was two words ('framing effect'). The mean number of words in response to this question was 26.4 and the median was 22.

### 4.1. Results from analyses of quantitative judgements

*Hypothesis 1: Round 2 judgements (post-discussion) will be more accurate than Round 1 judgements (pre-discussion) (at the participant level).*

**Figure 4.**
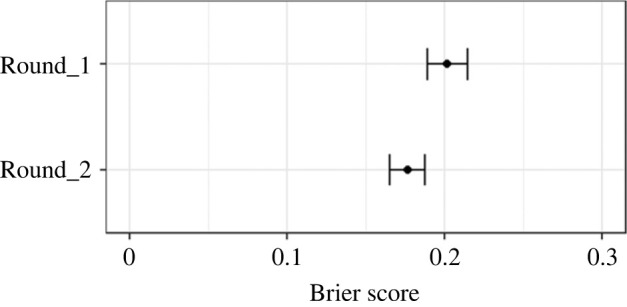
Average Brier scores per participant were lower (less error, more accurate) in Round 2, after discussion than in Round 1, before discussion. ‘Inference by eye’ indicates that this difference is statistically significant, with little or no overlap of 95% CIs between rounds.

Average Brier Scores (judgement error) were statistically significantly lower in Round 2 (after discussion) than in Round 1 (before discussion), reduced by 0.03, 95%CI [0.01, 0.04], *p* < 0.001. (figure 4). This effect size should be interpreted in the context of the average Brier scores for participants; the range being 0.127–0.251 for Round 1 to 0.123–0.236 for Round 2. In terms of raw estimates, this means that twice as many best estimate judgements improved (254) from Rounds 1 to 2 and got worse (121), while 250 stayed the same (i.e. did not change). Judgements that improved in Round 2 shifted 13% points in the right direction on average, while those that got worse shifted 11% points in the wrong direction, on average. At the participant level, 20 out of 25 individuals (80%) were more accurate in Round 2, averaged across the 25 claims. Those individuals who improved in Round 2 shifted 8% points on average, while those who got worse shifted 6% points (not a statistically significant difference for shift size). Overall, these results provide support for the beneficial impact of feedback and discussion on predictive accuracy when judging the replicability of claims in the social and behavioural sciences.

*Hypothesis 2: Those who perform well on a quiz testing familiarity with previous research and concepts related to assessments of replicability will be more accurate on the main task (at the participant level).*

Participants’ overall quiz scores were weakly and not statistically significantly correlated with the accuracy of their Round 1 estimates (Spearman *ρ* = −0.339, 95%CI [−0.647, 0.065], *p* = 0.098), and more strongly correlated with the accuracy of their Round 2 estimates (*ρ* = −0.458, 95%CI [−0.722, −0.076], *p* = 0.021). However, the modelling results showed that this correlation corresponds to only a small change in accuracy even in Round 2, where the correlation was strongest: each correct answer on the quiz reduced the participant's Average Brier score by approximately 0.006 [−0.010, −0.003]. Models showed that IDEA groups (included as a random effect) had a near-zero effect on accuracy in both rounds.

In an additional exploratory analysis, we examined the two main components of the quiz separately. Results for the statistical concepts component (out of 12 possible points) show a similar, marginally stronger correlation in Round 1 as the full quiz (*ρ* = −0.356, 95%CI [−0.659, 0.045], *p* = 0.080), and a stronger, statistically significant correlation in Round 2 (*ρ* = −0.516, 95%CI [−0.757, −0.152], *p* = 0.008). The model outputs (with a random effect for ‘group’) tell a similar story, with the statistics component quiz score showing a non-significant effect on accuracy in Round 1, and a significant (*p* < 0.01) effect of a small magnitude in Round 2 (each correct answer reduces Brier score by 0.009, 95%CI [−0.014, −0.003]).

The meta-research knowledge quiz component (out of 10 possible points) had non-significant, even weaker correlations with accuracy in both Round 1 (*ρ* = −0.189, 95%CI [−0.546, 0.226], *p* = 0.365) and Round 2 (*ρ* = −0.242 [−0.585, 0.176], *p* = 0.245). We suspected that performance on the statistical concepts quiz component might carry the most weight in the relationship with accuracy because these items required statistical knowledge or reasoning that can be more readily applied to the assessment of any claim. The meta-research items, on the other hand, target participants' memory of published replication success rates or average statistical power from previous meta-research projects. While knowledge of those base-rates arguably helps form informative priors for participants’ judgements, they do not discriminate performance as well as statistical reasoning.

*Exploratory Question 1: What are the demographic, experience and psychometric characteristics of (i) more accurate judges and (ii) those who update their judgements most between rounds?*

As anticipated, we did not detect many meaningful or statistically significant relationships between demographics, experience and training variables with judgement accuracy (we report on Round 1 here only, since we are more interested in the accuracy of individuals before discussion). We did, however, find a relationship between an individual's experience with pre-registration and their accuracy (*F*_1,23_ = 9.931, *p* = 0.004). We also detected a possible relationship between numeracy experience and accuracy. For two relevant variables, this relationship with accuracy was statistically significant (i.e. stats/quantitative courses taken, *N* = 25, *ρ* = −0.401, 95%CI [−0.687, −0.007], *p* = 0.047; BN scores, *N =* 23*, ρ =* −0.424, 95%CI [−0.721, 0.004]*, p =* 0.044). For another relevant variable, self-reported technical expertise in statistics, the Spearman correlation was also relatively high but not statistically significant (*ρ* = –0.371, 95%CI [−0.668, 0.029], *p* = 0.068, although note that this relationship was statistically significant using a linear model and ANOVA, *F*_1,23_ = 5.058, *p* = 0.034). Recall that Brier scores are a measure of judgement error, so negative correlation coefficients indicate that as the characteristic goes up (psychometric test scores, etc.), the error goes down.

Results from the other standardized tests, i.e. the CRT and the AOT, did not yield particularly strong results. We saw statistically significant relationships between the two numeracy-like scales (CRT and BN, *ρ* = 0.45, 95%CI [0.04, 0.73], *p =* 0.03), and participants who scored higher on the CRT questions—which is designed to measure a person's tendency to take the time to think and reflect before settling on an answer—also tended to update their judgements more after feedback and discussion (*ρ* = 0.47, 95%CI [0.07, 0.74], *p* = 0.02). Results from the AOT test lacked variation (everyone scored highly, i.e. ‘actively open minded’). Accordingly, we did not detect any relationships involving that variable.

In terms of updating judgements, we did not detect any other meaningful or statistically significant relationships. Results from these analyses can be found in electronic supplementary material, table S1.

*Exploratory Question 2: Are those who update (shift) their judgements more between rounds more accurate in Round 2 (after discussion, across all claims)?*

Those who shift the most on average not only improve the most between rounds (*ρ* = 0.50, 95%CI [0.13, 0.75], *p* = 0.01), but are also the most accurate in Round 2 (lower Brier score error, on average, *ρ* = −0.52, 95%CI [−0.76, −0.16], *p* = 0.008). These exploratory results are also supported by equivalent models that account for variation between groups (electronic supplementary material).

*Hypothesis 3: Those who update (shift) their judgements most between rounds will have (i) lower mean claim-level expertise ratings and (ii) lower mean claim-level understanding ratings (at the participant level).*

(i) Claim-level expertise: we detected that when expertise ratings are higher, the amount that a participant shifts their best estimate judgements after the discussion is reduced (*β* = −0.010, 95%CI [−0.019, −0.001], *t*(18.958) = −2.257, *p* = 0.036), but by a small magnitude. Each unit increase in average expertise (on the 1–7 scale) reduces the participant's average shift by approximately 1.025 [0.12, 1.90] (on a 0–100 scale, where the average size of shift for any given participant ranges from 3 to 15% points). We saw a smaller and statistically non-significant difference in the same direction for interval width shifting (*β* = −0.004, 95%CI [−0.015, 0.008], *t*(19.559) = −0.683, *p* = 0.503).
(ii) Claim-level understanding: understanding ratings were associated with inconsistent effects on the extent of shifting, with statistically non-significant effects for both best estimate and interval width. We refrain from interpreting these results further as the variation in understanding responses is too small to properly investigate this question. Everyone appeared to have a good understanding of the claims, ranging from 4.9 to 6.7 in Round 1, and 5.3 to 6.9 for Round 2 (on a scale of 1–7).

The following examples of qualitative reasoning help contextualize the discussion of the different roles played in influencing participant confidence and updating between rounds in this study. In the first example, one individual shifted their best estimate of replicability from 55 to 25, noting that:

*Discussion strengthened my beliefs about p-hacking, lack of pre-study planning, selective reporting and other QRPs. I find the underlying premise unlikely and anticipate there are a number of methodological issues.*

In this example, it is clear that discussion improved the individual's confidence in their original assessment of the claim. Another individual analysing the same claim also shifted their best estimate from 75 to 40, noting instead that the discussion highlighted aspects of the claim they had not fully considered:

*During the discussion, I heard … some counter-evidence for the premise of the study. Also, I learned that they [the authors] used an existing data set, and ran multiple tests, etc. All these got me worried a bit. The stats still looks good* [sic] *to me but I lowered my estimates in general.*

The final example included here illustrates the most common role of discussion in correcting or clarifying the claim being evaluated. This individual updated their best estimate from 70 to 50 between rounds, explaining that the discussion clarified a critical aspect of the study design and operationalization:

*… So I missed a very important point while making my first judgment. I think I now understand the claim better after the discussion. There was a mismatch between the statistics (sample size vs. reported df) and I did not realized* [sic] *this at the beginning. Small sample size but strong effects, I still think that this might replicate but I also accept that there is good chance that it won't.*

*Hypothesis 4: Judgements made on particular claims (i.e. not aggregated across claims for that participant) will be more accurate when associated with (i) higher claim-level expertise ratings, (ii) higher claim-level understanding ratings and (iii) more certain (narrower) intervals.*

We found a small and not statistically insignificant (*β* = −0.009, 95%CI [−0.019, 0.0001], *t*_225.17_ = −1.937, *p* = 0.054) effect of claim-level expertise on the accuracy, with higher claim-level expertise ratings associated less error, i.e. with more accurate judgements. We found a larger and statistically significant effect (*β* = 0.238, 95%CI [0.133, 0.341], *t*_564.867_ = 4.676, *p* < 0.001) of uncertainty on the accuracy, where wider intervals (i.e. less certain intervals) were associated with decreased accuracy. So, at a judgement level, in Round 1, a 10% increment difference in interval width would suggest a best estimate score that is approximately 2.4% [1.3, 3.4] (on the 0–100 scale) further away from the (correct) certainty limit. These results do not hold at the participant level (i.e. averaging claim-level expertise and judgement-level interval width across claims assessed by a given individual). And as above in Hypothesis 3, we found no effect of **‘**understanding’ ratings on accuracy, but see above for our concern over the lack of variation in this measure. On the whole, interval width seems to provide a better measure of confidence/uncertainty than other self-ratings that might signal confidence, i.e. expertise and understanding, even when the latter are elicited at specific claim-level.

Below are a variety of examples showing ways in participants' express confidence in their estimates, discussing notions of expertise, certainty and understanding. In the first example below, the participant is clear that the increased certainty they felt following discussion had been translated into narrower bounds in Round 2:

*I still don't think it will replicate because it is a very small effect (thought it would be nice to see what happens with an adequate sample size), though I moved my estimate up a bit to account for my better understanding of the proposed mechanism. But I feel less uncertain after the discussion so my bounds are smaller.*

In other cases, participants expressed having been generally swayed towards another member(s) estimates:

*Other people in the group had more knowledge in this area than me and they expressed some more confidence in the design so it raised my lower bound a bit.*

*I brought up my best and lower estimates after another group member explained the study to me better.*

There were also several examples where participants’ confidence had clearly been impacted by discussion but their comments did not explicitly identify how this was translated (or not) into updated estimates.

*My group had a lot more information about this type of research, and it made me a little more confident in this effect (slightly stronger prior).*

*Really influential to hear another group members say she's observed this pattern multiple times in her own data.*

For some, discussion reinforced participants' confidence in their own initial estimates.

*the arguments of the rest of the group against the claim seemed to be mostly methodological criticism. But as the replication would be conducted using the same methodology I don't think these arguments speak against a successful replication. The arguments in favor of a successful replication pretty much agree with my thoughts on this, so I feel more confident now.*

*Exploratory Question 3: Do participants with (i) lower average claim expertise ratings and (ii) lower average claim understanding ratings (across claims) become more accurate after discussion?.*

Additional analyses suggest that people with lower self-rated expertise (averaged per claim) improved the most between rounds (became more accurate, where negative values signify improvement, *ρ* = 0.53, 95%CI [0.17, 0.76], *p* = 0.007). We did not find a statistically significant relationship between self-rated claim understanding (averaged per claim) and improvement. Again, these exploratory results are robust to different statistical approaches, with equivalent models that account for variation between groups finding consistent results (electronic supplementary material).

### 4.2. Results from analyses of qualitative reasoning

**Figure 5.**
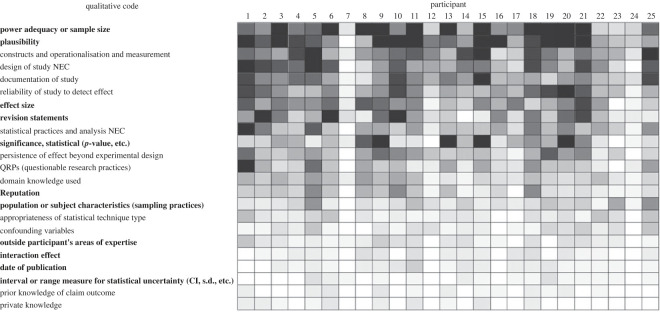
Summary of qualitative coding results. Participants are ordered left to right from most to least accurate by Round 2 Brier score. Codes are ordered from top to bottom by most to least frequently used overall. Codes that met the ICR threshold are shown in bold. NEC, not elsewhere categorized.

A summary of how participant responses were coded across the entire dataset can be seen in figure 5. This indicates how frequently each code was applied to a given participant's responses, with more frequent coding indicated by darker shading. For example, participant six was coded as including ‘revision statements’ in all 25 claims. In this figure, participants are ordered by Round 2 Brier score from left (most accurate) to right (least accurate), and codes are ordered by overall frequency of use from the top (most frequent) to bottom (least frequent). In this figure and other descriptive statistics presented below, we include all codes within the codebook but indicate (through bolding) which codes met the minimum ICR target. It should be noted that as the frequency of a code in the dataset decreases, the ICR score becomes more volatile; even a single accidental ‘miss’ by an analyst can make a large difference to ICR for a low-frequency code. Thus even codes that do not meet the ICR can be meaningful but they should be interpreted with caution.

*Exploratory Question 4: Are those who use a greater breadth of reasons to support their assessments also more accurate?*

**Figure 6.**
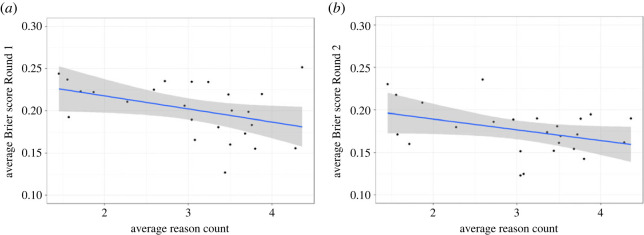
Relationship between breadth of reasoning (average reason count) displayed by participants (summed across both rounds) and their accuracy predicting replicability (lower Brier score is better). Round 1 (left) is of the most interest since this reflects pre-discussion accuracy. Round 2 (right) is also shown, for completeness.

Our exploratory qualitative analyses suggest that those who provided a greater number of unique reasons to support their assessments (summed across both rounds) were also more accurate in predicting replicability, as indicated by lower Brier scores (figure 6), although the relationship is weak in this dataset (Round 1 *R*^2^ = 0.17, *p* = 0.04, Round 2 *R*^2^ = 0.15, *p* = 0.06).

*Exploratory Question 5: Which reasoning categories are more commonly used by more accurate participants?*

**Figure 7.**
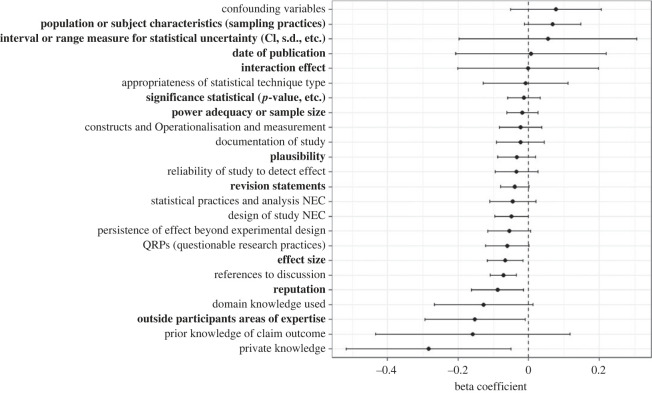
Relationships between reasoning codes and participant accuracy (reasoning codes to the left of the dashed line (*β* = 0.0) are used more often by more accurate participants (i.e. those with lower Brier scores, on average). Codes that met the ICR threshold are shown in bold. NEC, not elsewhere categorized. Error bars denote 95% CIs.

A number of reasoning codes were more commonly invoked by more accurate participants (although we caution that this analysis was exploratory). Codes that speak to the actual research claim include ‘effect size’ (*β* = −0.066, 95%CI [−0.120, −0.013], *t*_23_ = −2.573, *p* = 0.017) and ‘reputation’ (of the discipline/research field, journal or authors) (*β* = −0.088, 95%CI [−0.166, −0.011], *t*_23_ = −2.352, *p* = 0.028). Other claim-relevant codes worth mentioning, but did not meet the ICR threshold include ‘Private knowledge’ (e.g. the participant's personal experience about methods commonly used in a research group) (*β* = −0.284, 95%CI [−0.530, −0.038], *t*_23_ = −2.385, *p* = 0.026), ‘design of study’ (*β* = −0.049, 95%CI [−0.099, 0.002], *t*_23_ = −2.004, *p* = 0.057), ‘QRPs (questionable research practices' (*β* = −0.061, 95%CI [−0.126, 0.004], *t*_23_ = −1.946, *p* = 0.064), ‘domain knowledge used’ (*β* = −0.128, 95%CI [−0.276, 0.021], *t*_23_ = −1.782, *p* = 0.088) and ‘persistence of the effect beyond experimental design’ (*β* = −0.055, 95%CI [−0.120, 0.009], *t*_23_ = −1.776, *p* = 0.089) (figure 7). Other notable codes more commonly used by more accurate participants relate more to the elicitation process, or the participant than to the research claim itself. These include ‘revision statements’ (i.e. comments that explicitly indicate that the participant has revised (or not) their view after discussion) (*β* = −0.040, 95%CI [−0.082, 0.002], *t*_23_ = −1.957, *p* = 0.063) and when the claim was ‘outside participant's area of expertise’ (*β* = −0.152, 95%CI [−0.303, −0.002], *t*_23_ = −2.099, *p* = 0.047). The remainder of the codes examined showed little or no relationship with accuracy (see electronic supplementary material).

As described in the codebook, some of our analytic categories included different finer-grained aspects as part of their definition. For example, the broader analytic category of ‘Reputation’ included aspects such as ‘institutional reputation’, ‘discipline and area of research reputation’, ‘author reputations’ and ‘journal reputation’. Examples of reasoning from participants include:

*I'm aware that some of the last author's work has been (successfully) replicated.* (Reputation/author reputation; private knowledge; domain knowledge used)

*Relies on very subtle priming. Priming literature is particularly suspect. Sample size and power very low. This result is likely noise or the result of p-hacking* (Reputation/discipline and area of research reputation; power adequacy or sample size; constructs and operationalisation and measurement; QRPs)

*I also looked at the year of publication, the journal, sample size, and affiliation of the authors*. (date of publication; Reputation/journal reputation; power adequacy or sample size; Reputation/institutional reputation)

Similarly, the ‘documentation of study’ code included ‘power analysis reporting’, ‘detail and transparency of documentation’, ‘availability of original materials or data’, and ‘inconsistencies in the reported methods or analysis' as possible aspects.

*The analysis looks fine. Lack of a priori power analysis reported and use of mTurk makes me much more skeptical.*

*Other problematic language within the article that are cues of little statistical knowledge. No evidence of a priori power calculation. High likelihood of optional stopping.*

Texts coded for ‘availability of original materials or data’ illustrate that ‘electronic supplementary material' was another aspect of the documentation that participants considered.

*This topic was not familiar to me and I an [sic]unsure if this will or will not replicate. I couldn't see supplemental material.*

*The paper and supplemental material is not clear and to me seem unorganized/messy/rushed paper.*

We note the large breadth of reasoning within pieces of some participants’ texts. While some responses have one code applied, others have 5+ codes applied (as illustrated in the following examples). Although some of the individual codes do not correlate with accuracy, those participants who demonstrated greater breadth of reasoning across all their responses were more likely to be accurate (figure 6).

*The claim seems really intuitive, so it makes sense that someone would find this effect*. (plausibility)

*I moderated my certainty a bit in the face of others' skepticism. Wish we knew more about the stimulus set–replication will depend a lot on whether the same images are used and the extent to which they were cherry-picked.* (revision statements; detail and transparency of documentation; constructs and operationalisation and measurement; QRPs; reliability of study to detect effect)

*How was competition operationalized? This effect seems plausible but might be sensitive to population and contextual factors. Undergrads have limited generalizability*. (constructs and operationalisation and measurement; plausibility; design of study NEC; persistence of effect beyond experimental design)

*Sample size is not that small, but the claim does not include other comparison run in the study (e.g. vs. males). Were p-values corrected for multiple testing?* (statistical practices and analyses; power adequacy or sample size; QRPs; appropriateness of statistical technique type)

*Lower: the predicted hypothesis seems a bit far fetch to me [sic], little info about the terms used in the stroop task. Upper: within-subject design, small p-value* (plausibility; constructs and operationalisation, and measurement; statistical practices and analyses; detail and transparency of documentation; design of study NEC; significance, statistical)

Codes were applied to text regardless of whether they indicate confidence or lack of confidence in the research claim, or neutrality. No attempt was made to assess the quality of reasoning within the coded text. The concepts underlying the qualitative codes could be invoked in a wide range of ways and needed to be understood in the context of the overall text. A good example of this is Effect Size, one of the codes where its use does appear to correlate with participant accuracy (figure 7). Depending on the overall claim, its prior plausibility and the study design, participants could understand a large effect size as making a claim more credible:

*Mostly swayed by how strong the effect is*. (effect size)

Or less credible:

*HUGE effect size that seems unrealistic. Like, wouldn't a bunch of people who've been to college know if it was so much better to study one way than the other*. (effect size, plausibility)

Similarly, small effect sizes could be interpreted as either positive or negative:

*Seems like it is a small effect (which I would expect). Small p-value. Huge sample size to be able to detect a small effect which makes me confident in replicability.* (effect size, statistical significance, sample size, replicability of claim)

*May not be relevant, but I don't find the result compelling – we're talking about a 0.39-point difference in the middle of a 7-point scale*. (effect size)

Some participants themselves were reflexive about this.

*It was interesting that many people seemed to trust the effect size in this, and even use it as a justification for why they thought it might replicate. I had the opposite reaction, i.e., the effect size was implausibly large and it made me lose confidence in this result*. (effect size, plausibility, reference to the discussion)

What is presented here is a snapshot of the possible qualitative analysis for this dataset. Here we have focused on the ‘breadth of reasoning’ and its relationship to accuracy. More is beyond the scope of this paper.

## 5. General discussion

Compared with the results of similar prediction markets and surveys, participants in our study were very good at predicting the replicability of research claims from social and behavioural sciences. Our prediction accuracy for these 25 studies was 84%, and prediction markets for the studies our claims were sampled from ranged from 71% to 86%. (Surveys of the same studies ranged from 58% to 86%). The large range in prediction market and survey results in part reflects changes in the methodology of conducting replication studies themselves. In earlier projects (e.g. reproducibility project: psychology), replication studies were on average not as high-powered as replication studies in more recent projects (social science reproducibility project). The set of claims we evaluated here span both earlier and more recent projects, but our sample only included claims for evaluation that were tested against a high-powered replication study (at least 90% power to detect an effect 75% of the size of that seen in the original study). This sampling strategy may partly explain why our relatively accurate results are more comparable to those achieved in later replication prediction projects (e.g. [14,16]) than earlier ones (e.g. [13]).

We acknowledge the small sample size of our study. This is a common shortcoming of studies involving groups, particularly deliberating groups, which requires a greater time commitment from participants. We also acknowledge that our participants were making ‘predictions’ retrospectively, so there is a possibility that some participants were aware of the published outcomes of some claims (see our Methods for how we attempted to buffer against this possibility, but note that there were 22 unique responses (of 1889 total participant comments) for which the ‘prior knowledge of claim outcome’ code was used by at least one of the three coders). A further limitation includes constraints on the generality of our results. Participants in our study assessed research claims from the social and behavioural sciences and possessed relevant expertise in that field. There is no guarantee that people, even with relevant expertise, would perform as well predicting replicability in other disciplines, where this may be more difficult to judge, e.g. due to the specialist and technical expertise required to grasp the methods, together with the insufficient detail on experimental protocols provided in the original paper, as found in preclinical cancer biology [11]. Nonetheless, the accuracy of our results supports the idea that structured elicitation methods, like the IDEA protocol, have a useful role to play in the evaluation of research [50].

### 5.1. Effect of updating and discussion on replicability judgements

The improvement we found in Round 2, following feedback and discussion, is generally consistent with findings of previous studies that used the structured IDEA protocol to elicit judgements. In our study, 80% of individuals moved towards the correct answer following feedback and discussion (Round 2). In the Hemming *et al.* [51] application of IDEA in environmental risk analysis, individuals moved towards the correct answer 67% of the time. We note that in Hemming *et al.* [51], shifts towards the correct answer were more substantial than shifts away from it, while this difference was small in this study (approx. 2% point difference in average shift size). Our results are also broadly compatible with those of the Good Judgement Project in geopolitical forecasting [52], which found that those who worked in teams, discussed and debated evidence and exchanged rationales were more accurate than those who worked alone. More recent studies provide further evidence that group interaction and discussion improve the accuracy of individuals [53], particularly when structured in small, independent groups [54], and when groups were already collectively well-calibrated (i.e. more accurate people were more confident, and less accurate people were less confident going into discussion) [55]. Under these conditions, the most knowledgeable (and confident) people were more likely to influence the answers of the less knowledgeable people in the group.

The results for the association between the application of specific reasoning codes and accuracy were exploratory only, and the results in any case weak, but it is notable that the strongest association was seen with the application of the code ‘revision statements’ (figure 7), which lends some support to the value of updating.

### 5.2. Participant expertise and prior knowledge

Given the wide CIs around the correlation between quiz scores and the accuracy of participants' Round 1 estimates, the overall quiz score did not serve as a convincing predictor of accuracy in this study. Having said that, exploratory analysis suggests more of a signal from the statistical concepts component of the quiz than the component testing familiarity with meta-research. This is something we will investigate in future research. Results from other analyses also provide evidence of a relationship between statistical literacy and performance when it comes to predicting the replicability of research claims, with numeracy test scores and self-reported expertise in statistics both showing moderate correlations with accuracy.

Our study did not detect relationships between accuracy and more traditional markers of expertise (i.e. background, education, publications, training and experience), again in exploratory analysis. This result was unsurprising and is in line with other research that has also failed to detect such relationships [21]. Interestingly, our exploratory qualitative analysis of participant reasoning indicated that more accurate participants overall (after discussion) were also more likely to articulate that research claims were outside their area of expertise.

Given the persistent challenges in determining, *a priori*, who are the best experts for forecasting and judgements, it was notable to find a statistically significant relationship between accuracy and one marker of expertise, namely, experience with pre-registration. Those who reported that they had ‘pre-registered at least one study’ were statistically significantly more accurate before discussion (i.e. Round 1, *F*_1,23_ = 9.931, *p* = 0.004). It is potentially useful to know that practical experience with preregistering research in advance, i.e. thinking through and transparently documenting the design and analysis plan for a study, may indicate an ability to accurately evaluate the quality (in this case, replicability) of other published research. Of course, this relationship may well disappear as pre-registration becomes more widespread. It is perhaps only indicative now because we are still in an ‘early adopters’ phase. More optimistically, it might suggest that training in pre-registration helps develop evaluation skills.

### 5.3. The relationship between confidence (including claim-level expertise) and accuracy

In this study, we explored the relationship between subjective confidence or uncertainty and accuracy in three different ways. For each claim, (i) participants rated their expertise in the domain of the claim, (ii) their understanding of the claim and (iii) they provided uncertainty in the form of interval bounds. We report above (Hypothesis 4) that higher expertise self-ratings for the specific claim domain were weakly associated with more accurate predictions. This is in the direction we would expect, but more research is needed to substantiate the relationship. We reiterate that although there appears to be a relationship here at the judgement level, there is no participant-level relationship between overall expertise self-ratings (averaged across claims) and average Brier scores for that individual. So, those who consider themselves to be more or less expert overall (on this set of claims), are no more or less accurate in judging replicability.

We did not detect a relationship between claim-level understanding and accuracy but see our previous comments about the lack of variation in participant's claim-level understanding scores. We did, however, find a relationship between interval width and accuracy at the judgement level, where best estimates accompanied by narrower intervals were more accurate than those accompanied by wider (less certain) intervals (again, this does not hold at the participant level). This relationship has been previously noted in eliciting intervals around quantities with a given level of confidence (e.g. [56,57]). The relationship is more complicated for probability judgements (which are bounded between 0 and 1) because probabilistic best estimates closer to the extremes (0 or 1) contain information about subjective confidence. In addition, a more extreme probability judgement is inherently more likely to be accompanied by a narrower interval due to the smaller distance to the nearest certainty limit, unless it is highly asymmetric. Nevertheless, participants may be using interval width to express subjective confidence.

### 5.4. ‘Breadth of reasoning’ as a potential proxy for accuracy

The results of the regression of the reasoning scores against Brier scores support our intuition that ‘breadth of reasoning’ is a useful proxy for performance in predictions, although these results need to be interpreted cautiously due to their exploratory nature. It should also be noted that there are significant individual variations, partly depending on how comfortable participants are with providing textual responses. In general, this textual dataset is undersampled. Textual responses were short in nature for a range of reasons. For Round 2, participants engaged in face-to-face discussions, which were typically more extensive than the text comments. Even in the best cases, participants provided a distilled version of their reasoning and reflections in their Round 2 comments. Indeed, some participants describe very little of their thinking (figure 6), and thus end up with low reasoning scores, despite being accurate predictors. In any case, we suggest that this measure is worth further investigation.

We note a limitation of the Reasoning score measure produced in this research arising from the calibration process. The decision to do two complete rounds of coding for this data, due to the relatively small size of the data, delayed final coding. While this enhanced the integrity of coding it also reduced the value of the second calibration meeting. The number of codes reaching the ICR threshold was lower than for other batches of coding done within the repliCATS project, with slightly different processes for coder calibration. Thus there may be slightly less signal in the Breadth of Reasoning scores produced for this study than would be possible using other coding processes. However, again, given the exploratory nature of this part of the research we make no strong claims here.

## 6. Conclusion

While the accuracy and uncertainty of individuals participating in this study varied, employing a structured elicitation protocol that allowed participants to learn from others overall resulted in more accurate predictions of replicability, comparable to the best results previously seen in prediction markets. Our approach also elicited detailed information about the reasoning and justifications participants gave for their judgements, which provides insight into how to further improve predictions and evaluation of research in the future. For example, successful reasoning strategies may inform peer review training, and be incorporated into future evaluation rubrics or checklists. The opportunity to receive feedback from the group, share information and update judgements are the critical components of our repliCATS approach and contributed to the accuracy of predictions in this study. We expect the accuracy of predictions to improve in the future, as elicitation methods are refined, and as the methodology of replication studies improves.

## Data Availability

Data (processed), code and materials: Open Science Framework, accession number pj3v8 (https://osf.io/pj3v8). Pre-registration: Open Science Framework, Project, Registration created August 21, 2019, accession number 5rj76 (https://osf.io/5rj76). Participant training materials for the broader repliCATS project: Open Science Framework, accession number a89nc (https://osf.io/a89nc/). Additional analyses and materials supporting this article have been uploaded as part of the electronic supplementary material [58].
